# Changes in Ascorbic Acid, Phenolic Compound Content, and Antioxidant Activity In Vitro in Bee Pollen Depending on Storage Conditions: Impact of Drying and Freezing

**DOI:** 10.3390/antiox14040462

**Published:** 2025-04-12

**Authors:** Rosita Stebuliauskaitė, Mindaugas Liaudanskas, Vaidotas Žvikas, Violeta Čeksterytė, Neringa Sutkevičienė, Šarūnė Sorkytė, Aurita Bračiulienė, Sonata Trumbeckaitė

**Affiliations:** 1 Department of Pharmacognosy, Faculty of Pharmacy, Lithuanian University of Health Sciences, Sukilėlių av. 13, LT-50162 Kaunas, Lithuania; rosita.stebuliauskaite@lsmu.lt (R.S.); mindaugas.liaudanskas@lsmu.lt (M.L.); sonata.trumbeckaite@lsmu.lt (S.T.); 2 Institute of Pharmaceutical Technologies, Faculty of Pharmacy, Lithuanian University of Health Sciences, Sukilėlių av. 13, LT-50162 Kaunas, Lithuania; vaidotas.zvikas@lsmu.lt; 3 Lithuanian Research Centre for Agriculture and Forestry, Institute of Agriculture, Instituto Ave. 1, LT-58344 Akademija, Lithuania; violeta.ceksteryte@lammc.lt; 4 Animal Reproduction Laboratory, Faculty of Veterinary Medicine, Lithuanian University of Health Sciences, Tilžės Str. 18, LT-47181 Kaunas, Lithuania; neringa.sutkeviciene@lsmu.lt (N.S.); sarune.sorkyte@lsmu.lt (Š.S.); 5 Laboratory of Biochemistry, Neuroscience Institute, Lithuanian University of Health Sciences, Eivenių Str. 4, LT-50162 Kaunas, Lithuania

**Keywords:** frozen and dried bee pollen, ascorbic acid, total phenolic content, flavonoids, storage time, antioxidant activity, antiradical and reducing activity

## Abstract

Bee pollen (BP) is a very valuable bee product, and its value depends on its proteins, lipids, amino acids, carbohydrates, vitamins, minerals, and biologically active compounds such as phenolic compounds, which may change depending on the method of pollen preparation after collection and its storage conditions. Therefore, it is very important to determine when the decline in bioactive compounds in BP occurs during storage. The purpose of this study was to evaluate the changes in the content of ascorbic acid and phenolic compounds, and to determine the antioxidant activity of BP extracts depending on their preparation method and storage conditions over a 15-month period, with assessments conducted every 3 months. Dried pollen (at +28 °C on the first day and +35 °C on the second day) and frozen (−20 °C and −80 °C) BP samples were prepared. After 3 months of storage, there was no decrease of ascorbic acid in frozen BP; however, it decreased by 20% in dried BP (*p* < 0.05). It was determined that in frozen BP, the content of total phenolic compounds decreased by 12–14% (*p* < 0.05) after 6 months, and in dried BP, it decreased by 7% (*p* < 0.05) after 3 months. The levels of flavonoids decreased by 10–17% (*p* < 0.05) in BP after 6 months. Chlorogenic and *p*-coumaric acids have been observed as the most abundant phenolic acids in BP. During storage (the 6–15-month period), the strongest antiradical and reducing activity in vitro was estimated in the frozen (−80 °C) BP, which was 1.8–3.4-fold and 2.6–3.1-fold higher, respectively, compared to the dried BP extracts. In conclusion, significant results were obtained, showing better stability of phenolic compounds and ascorbic acid during storage in frozen BP compared to dried pollen. Melisopalynological analysis revealed a polyfloral pollen mixture, with *Salix* spp. and *Brassica napus* L. predominating in all samples, comprising 34.3% and 36.8%, respectively. Among these, *Acer platanoides* L., *Malus domestica* Borkh., and *Taraxacum officinale* L. were important minor pollens present in the samples examined.

## 1. Introduction

Bee pollen (BP), consisting of flower pollen grains, nectar, and salivary secretions of bees, is a highly valuable bee product. Bee pollen is known for its anti-inflammatory [1,2], antibacterial [3], antidiabetic [4,5,6], anticancer [7,8], and other effects. Its nutritional and medicinal value is determined by its amounts of proteins, lipids, amino acids, carbohydrates, vitamins, minerals, and various biologically active compounds such as enzymes and phenolic compounds [9,10]. Among the many biological effects on the body, antioxidant activity research has received the most attention. BP exhibits strong antioxidant activity, which is primarily ascribed to the diversity of phenolic compounds, ascorbic acid, and tocopherol [1,11,12,13,14,15,16]. In recent decades, compounds with antioxidant properties have become a target for scientific studies, as there is a constant search for new sources capable of combating oxidative stress [17,18].

The primary challenge in the use of BP in modern phytomedicine is associated with the variability in phytochemical composition, which depends on various factors, including the botanical and territorial origin of the pollen, natural conditions, and especially the preparation and storage conditions of the raw material [19,20]. To ensure the production of high-quality BP products, it is essential to guarantee not only suitable preparation conditions but also optimal storage conditions. These directly influence the phytochemical composition and biological activity of BP, as well as its potential applications as a functional food [20,21].

To maximize the retention of vitamin C, polyphenols, and antioxidant properties in bee pollen, freezing (−20 °C) is the most effective method, while drying at 40 °C results in significant degradation of these bioactive compounds. Dry pollen is usually provided to consumers in Lithuania, but its phytochemical composition and quality are highly dependent on the drying temperature [22]. Unfortunately, the drying process, especially at higher temperatures, can result in a significant reduction in the amount of biologically active compounds, including vitamins, which are particularly susceptible to high temperatures [23]. The degradation of ascorbic acid and phenolic compounds in bee pollen during storage can be influenced by several factors. Oxidation is one of the key pathways, especially for ascorbic acid, which is highly sensitive to oxygen, light, and temperature. Ascorbic acid is reversibly oxidized to dehydroascorbic acid (DHA) under light, heat, metal ions, and alkaline pH, which then irreversibly degrades to 2,3-diketogulonic acid [24]. Enzymatic activity, particularly from polyphenol oxidase, may also contribute to the degradation of phenolic compounds when enzyme inactivation is incomplete, especially in dried pollen [25]. Heat during drying may cause thermal decomposition of flavonoids and vitamin C, while even frozen samples are susceptible to slow oxidative processes over extended periods. 

Pollen that is freshly collected and frozen immediately after collection is very popular in the foreign market [26]. To date, comprehensive studies systematically analyzing changes in polyphenolic compounds, vitamin C, and antioxidant activity of bee pollen during extended storage—particularly at regular 3-month intervals over a 15-month period—are lacking. Such data are essential for evaluating the suitability of bee products for consumption and their capacity to retain bioactive compounds over time. In order to introduce a preparation method for BP that allows for the production of high-quality pharmaceutical preparations, functional foods, or dietary supplements, or to consume high-quality BP in its native state, it is essential to study the differences in the phytochemical composition between dried and frozen BP and to determine the variations in the phytochemical composition of BP during storage [23,27]. Since only the highest quality BP must reach consumers, the requirements for their quality are increasing.

Therefore, the aim of our study was to evaluate the changes in the composition of phenolic compounds and ascorbic acid of bee pollen samples and to determine antiradical and reducing activity in vitro of bee pollen extracts depending on preparation methods (freezing or low temperature drying) over a 15-month storage period, with changes assessed every three months. This study was oriented toward consumer guidance, aiming to provide practical recommendations regarding how long bee pollen can be stored under typical conditions before significant degradation of key bioactive compounds begins. To date, the scientific literature lacks detailed studies that comprehensively examine these parameters over such a long storage duration, making our findings particularly novel and significant in the field of phytochemical stability.

## 2. Materials and Methods

### 2.1. Collection of Pollen and Estimation of Its Botanical Origin

Bee pollen (BP) was collected from an apiary located in Talkoniai, Pasvalys district in 2024 in April–May. The location coordinates are 55.9598° N, 24.3422° E. Pollen loads were collected with pollen traps in good weather during peak plant blooming periods of about 200 g/per day. Pure, fresh bee-collected pollen was cleaned and distributed for storage according to the research conditions described below. During the research, freshly collected and immediately frozen and dried bee pollen samples were examined.

### 2.2. Preparation of Bee Pollen

#### 2.2.1. Preparation of Dried Bee Pollen Samples

Freshly collected BP samples were dried in a dryer, maintaining a temperature of +28 °C for 24 h and +35 °C on the second day for 24 h. The prepared raw material was stored in a sealed container in a dry, dark, well-ventilated room at room temperature (+19–21 °C) throughout the entire study.

#### 2.2.2. Preparation of Frozen Bee Pollen Samples

Freshly collected BP samples were immediately frozen at −20 °C and −80 °C in freezers. They were kept in sealed containers at a constant temperature (−20 °C and −80 °C, respectively) throughout the study. The extracted raw material was immediately processed. Variations in the qualitative and quantitative composition of the BP were studied every three months. All results were recalculated to the dry weight.

### 2.3. Microscopic Examination of Pollen Samples

BP granules were evaluated according to morphological characteristics such as color and shape [28]. Pollen sample preparation for botanical composition analysis was performed using the melissopalynology technique described by Louveaux et al. [29]. Pollen photos taken using a Nikon Eclipse E600, model C-LP (Nikon Corporation, Tokyo, Japan) microscope were compared with the known plant pollen photos presented in a pollen catalogue [30].

Pollen expression results: About 400–500 pollen grains were counted in each sample. The frequency of pollen grains of each melliferous plant is expressed as a percentage of the total pollen sum. Pollen considered as monofloral was mainly produced from one plant species, or pollen content from one plant species was predominant (constituting more than 45%). The pollen content of other plant species was designated as follows: secondary pollen, 16–45%; important minor pollen, 3–15%; minor pollen. All analyses were carried out in triplicate. Estimated values are expressed as means ± standard deviations (SDs) and the coefficient of variance (CV).

### 2.4. Chemicals

All solvents, reagents, and standards used were of analytical grade. The solvents used were as follows: purified water (Millipore^®^, Bedford, MA, USA), ethanol 96.0% (*v*/*v*) (AB Vilnius degtinė, Vilnius, Lithuania).

The reagents used were as follows: ≥99% gallic acid monohydrate, ≥99% Folin–Ciocâlteu reagent, ≥99% sodium carbonate, ≥99% hexamethylenetetramine, ≥99% acetic acid, ≥99% aluminum chloride hexahydrate, ≥98% Trolox^®^ (6-hydroxy-2,5,7,8-tetramethylchroman-2-carboxylic acid), ≥99% ammonium acetate, ≥98% TPTZ (2,4,6-tri-(2-pyridyl)-1,3,5-triazine), ≥37% hydrochloric acid, ≥99% sodium acetate trihydrate, ≥99% glacial acetic acid (Sigma-Aldrich^®^, Steinheim, Germany), ≥98% rutin standard (Extrasynthesis, Genoa, Italy), ≥98% ABTS (2,2′-azino-bis-(3-ethylbenzthiazoline-6-sulfonic acid)) (TCI Europe, Zvijndrecht, Belgium), ≥98% potassium persulfate (Alfa Aesar GmbH and Co KG, Kandel, Germany), ≥98% neocuproine, ≥98% copper (II) chloride dihydrate, ≥98% iron (III) chloride hexahydrate, ≥98%sodium 2,6-dichlorophenolindophenolate, ≥99% chloral hydrate (2,2,2-trichloroethane-1,1-diol) (Roth, Karlsruhe, Germany).

The standards of phenolic compounds used were as follows: ≥98% *p*-coumaric acid, ≥99% gallic acid, ≥98% ferulic acid, ≥98% rosmarinic acid, ≥98% chlorogenic acid, ≥98% kaempferol, ≥98% kaempferol-3-O-glucoside, ≥98% kaempferol-3-O-rutinoside, ≥98% quercetin, ≥98% hyperoside, ≥98% quercetin-3-arabinopyranoside, ≥98% rutin, ≥98% ≥98% isorhamnetin-3-glucoside, ≥98% isorhamnetin-3-rutinoside, ≥98% luteolin, ≥98% luteolin-4-O-glucoside, ≥98% luteolin-3,7-diglucoside, ≥98% phloridzin, ≥98% phloretin, ≥98% apigenin, ≥98% naringenin, ≥98% isoquercitrin, ≥98% isorhamnetin, ≥98% avicularin, ≥98% quercitrin (Sigma-Aldrich^®^, Steinheim, Germany).

### 2.5. Preparation of the Bee Pollen Extracts

#### 2.5.1. Preparation of Ethanol Bee Pollen Extract

The methodology for the preparation of ethanol bee pollen extract was described by Kahraman et al. [31]. A total of 1 g (exact weight) of crushed, dried, and frozen (−20 °C and −80 °C) BP was weighed. The weighed amount of the raw material was transferred to labeled vials made of dark glass with the same volume, and 10 mL of 70.0% (*v*/*v*) ethanol was added. Three vials of each raw material (n = 9) were prepared and exposed to an ultrasonic bath for 50 min. Afterward, solid particles were separated from the liquid phase using a vacuum pump connected to a filtration system, with the liquid portion collected to an exact volume of 10 mL. The raw material residue on the filter was washed with a 70.0% (*v*/*v*) ethanol–water mixture. The obtained ethanol extracts were poured into dark glass sealed bottles, labeled, and stored at room temperature in a dark place until the next step. The prepared extracts were used to identify and determine the amounts of phenolic compounds and assess the antiradical and reducing activity in vitro.

#### 2.5.2. Preparation of Aqueous Bee Pollen Extracts

The methodology for the preparation of aqueous bee pollen extract was described by Ares et al. [32]. A total of 2.5 g (exact weight) of crushed dried and frozen (−20 °C and −80 °C) BP was weighed. The weighed amount was transferred to labeled conical flasks of equal volume (100 mL), poured with 25 mL of purified water, and left for 10 min to extract three samples of each raw material (n = 9). Using a vacuum pump with a connected filtration system, the solid particles from the liquid phase to an exact volume of 25 mL were separated, washing the residue of the raw material on the filter with purified water. The obtained aqueous extracts were used for the estimation of ascorbic acid in the BP samples.

### 2.6. Evaluation of Total Phenolic Compounds (TPC) and Flavonoid Contents in Bee Pollen Samples

All spectrophotometric measurements were carried out using a HALO DB–20 UV–VIS spectrophotometer (Dynamica GmbH, Salzburg, Austria). Total phenolic content was determined using the Folin–Ciocâlteu method in the ethanolic extracts of BP [33]. The light absorption of a 10 mm layer of the analyzed solution was measured at a wavelength of 765 nm. The calibration curve equation was y = 0.4016x + 0.0148 (R^2^ = 0.9985). The results were determined based on the standard curves of gallic acid prepared in the range of 0.03–1.0 mg/L and expressed as milligrams of gallic acid equivalent (GAE) per gram of dry sample weight (mg GAE/g DW). The total flavonoid content in the ethanolic BP extracts was estimated using the methodology described by Urbonavičiūtė et al. [34]. The absorption of the analyzed solution was recorded at a wavelength of 407 nm. The calibration curve equation was y = 0.503x − 0.0141 (R^2^ = 0.9954). The results were calculated based on a rutin calibration curve and expressed as milligrams of rutin equivalent (RE) per gram of dry sample weight (mg RE/g DW).

### 2.7. Determination of Ascorbic Acid in Bee Pollen Samples Using the Titrimetric Method

A total of 1 mL of the prepared BP aqueous extract, 1 mL of hydrochloric acid (20 g/L solution), and 13 mL of purified water were added to a 50 mL conical flask. The resulting mixture was titrated with a 0.001 M solution of sodium 2,6-dichlorophenolindophenolate in a microburette until a pale pink color persisted for 10 s. The titration was performed three times, and the arithmetic mean was calculated. A total of 1 mL of a 0.001 M solution of sodium 2,6-dichlorophenolindophenolate corresponded to 0.000088 g of ascorbic acid. The amount of ascorbic acid for the dry raw material was calculated using the following formula:

X = (V × 0.000088 × V_1_ × 100 × 100)/(m × V_2_ × (100 − W))

X—amount of ascorbic acid, %;

V—amount of sodium 2.6-dichlorophenolindophenolate 0.001 M solution used for titration, mL;

V_1_—volume of the research extract, mL;

V_2_—test extracts taken for titration, volume, mL;

m—weighed raw material mass, g;

W—loss on drying, %.

### 2.8. Quantitative and Qualitative Determination of Phenolic Compounds in Bee Pollen Samples Using the UHPLC–MS/MS Method

To qualitatively and quantitatively evaluate the individual phenolic compounds in the BP samples, a validated UHPLC–MS/MS method was employed [35]. The separation of phenolic compounds was carried out using an Acquity H-class UPLC system (Waters, Milford, CT, USA) coupled with a triple quadrupole tandem mass spectrometer, Waters Xevo TQD (Waters, Milford, CT, USA), equipped with an electrospray ionization (ESI) source for MS/MS data acquisition. A YMC Triart C18 column (100 × 2.0 mm; 1.9 μm) (YMC Europe GmbH, Dinslaken, Germany) was used for the analysis. The column temperature was maintained at 40 °C. Gradient elution was performed with a mobile phase consisting of 0.1% formic acid aqueous solution (solvent A) and acetonitrile (solvent B) at a flow rate of 0.5 mL min^−1^. A linear gradient profile was applied as follows for solvent A: initially 95% for 1 min, reduced to 70% over 4 min, further decreased to 50% over 7 min, and returned to 95% over 2 min. Negative electrospray ionization was used for the analysis, with the following parameters: capillary voltage, −2 kV; source temperature, 150 °C; desolvation temperature, 400 °C; desolvation gas flow, 700 L h^−1^; cone gas flow, 20 L h^−1^. The collision energy and cone voltage were optimized individually for each compound (Table 1).

### 2.9. Determination of Antiradical and Reducing Activities In Vitro in Bee Pollen Extracts

The antioxidant activities of the BP extracts were established based on three different antioxidant capacity assays, namely in vitro spectrophotometric ABTS free radical scavenging and ferric (FRAP) and copper (CUPRAC) reducing activity assays using a spectrophotometer HALO DB–20 UV–VIS Dynamica (Dynamica GmbH, Salzburg, Austria).

#### 2.9.1. Evaluation of Antiradical Activity Using the ABTS Method In Vitro

The antiradical activity was measured using a method modified from Khongkarat et al. [36]. The stock and working solutions were prepared as follows: a 2 mmol/L ABTS^●+^ stock solution was prepared by dissolving 0.0548 g of ABTS in 50 mL of purified water, then adding 0.0095 g of K_2_S_2_O_8_. The solution was kept in the dark for 16 h to form the ABTS^●+^ radical, which remained stable for two days at room temperature. The working solution was obtained by diluting the stock until absorbance at 734 nm (λ) reached 0.800, measured with a 10 mm path length. Sample preparation: 10 μL of the ethanol extract was mixed with 3 mL of the working ABTS^●+^ solution. Samples were incubated for 30 min in the dark at room temperature, and the color change from blue-green to pale blue was observed. Absorbance at 734 nm (λ) was measured. A calibration curve (y = 0.00007x + 0.088; R^2^ = 0.9932) was created using Trolox standards (125–8000 μmol/L).

#### 2.9.2. Estimation of Reducing Activity Using the CUPRAC Method In Vitro

The method for determining reducing activity, as described by Munteanu et al. [37], was applied. Preparation of stock solution: solutions A, B, and C were mixed in a 1:1:1 ratio. Solution A: 10 mmol/L CuCl_2_×2H_2_O (0.085 g in 50 mL of purified water). Solution B: 1 mmol/L CH_3_COONH_4_ buffer (pH 7, 0.0038 g in 50 mL of purified water). Solution C: 7.5 mmol/L neocuproine (0.078 g in 50 mL of 96.0% ethanol). Sample preparation: 10 μL of the ethanol extract was mixed with 3 mL of freshly prepared working solution. The samples were incubated in the dark at room temperature for 30 min, with a color change observed from light blue to orange-yellow. Absorbance at 450 nm (λ) was measured using a spectrophotometer with a 10 mm path length. A blank was prepared using the working CUPRAC solution. A calibration curve (y = 0.00004x + 0.0047; R^2^ = 0.9998) was constructed using Trolox standards (125–32,000 μmol/L).

#### 2.9.3. Evaluation of Reducing Activity Using the FRAP Method In Vitro

The method for determining reducing activity, as described by Sawicki et al. [3], was applied. Preparation of working solution: solutions A, B, and C were mixed in a 1:1:10 ratio. Solution A: 20 mmol/L FeCl_3_×6H_2_O aqueous solution (0.2704 g of FeCl_3_ dissolved in 50 mL of purified water). Solution B: 10 mmol/L TPTZ solution (0.1562 g of TPTZ dissolved in 50 mL of purified water acidified with 40 mmol/L HCl). Solution C: 300 mmol/L acetate buffer (CH_3_COONa×3H_2_O solution, 0.775 g of CH_3_COONa×3H_2_O mixed with 4 mL of glacial acetic acid, diluted to 250 mL with purified water). Sample preparation: 10 μL of the ethanol extract was mixed with 3 mL of the working solution. The samples were incubated in the dark at room temperature for 30 min, observing a color change from colorless to intensely blue. Absorbance was then measured at 593 nm (λ) using a spectrophotometer with a 10 mm path length. A blank was prepared using the working FRAP solution. A calibration curve (y = 0.00003x + 0.0233; R^2^ = 0.9932) was constructed using Trolox standards (500–32,000 μmol/L).

#### 2.9.4. Assessment of Antioxidant Activity In Vitro

The antioxidant activity of the BP extracts was calculated based on Trolox calibration curves and expressed as the Trolox equivalent (μmol TE/g) per absolute dry weight. The Trolox equivalent corresponds to the amount that, under the same testing conditions, exhibits the same antioxidant activity as 1 g of BP raw material [37,38]. In vitro antioxidant activity was calculated using the following formula:

TE = (A + b)/(a × 1000) × (V_sample_)/(m_sample_)

A—absorbance of the sample;

a—slope of the Trolox calibration curve equation;

b—deviation from the Trolox calibration curve equation;

V_sample_—volume of BP extract, mL;

m_sample_—weight (precise) of BP powder, g.

### 2.10. Statistical Analysis

To evaluate the results obtained during this research, statistical data analysis was performed using Microsoft Office Excel 2013 (Microsoft, Redmond, WA, USA) and SPSS Statistics 29 (SPSS Inc., Chicago, IL, USA). The experiments were repeated in triplicate, with the results presented as arithmetic means ± standard deviations (SDs) of replicates. The variables were calculated and graphically presented using Microsoft Office Excel 2013, and the SPSS Statistics 29 package was used to assess whether differences between the obtained values were statistically significant. One-way analysis of variance (ANOVA) was applied using Tukey’s Post Hoc test (significance level *p* < 0.05). The correlation between the content of total phenolic compounds, total flavonoids, the sum of individual phenolic compounds, ascorbic acid, and antioxidant activity (measured using ABTS, CUPRAC, and FRAP methods) was expressed by Pearson’s correlation coefficient. The values of the Pearson’s correlation coefficients were selected and applied based on the values provided by Šimoliūnienė R. et al. [39].

## 3. Results

### 3.1. Changes in Total Phenolic Compounds (TPC) and Flavonoid Contents in Bee Pollen Samples

In order to assess changes in the composition of phenolic compounds and ascorbic acid in the BP samples after different preparation methods (freezing at −20 °C and −80 °C, or drying at low temperature) and during storage, the total contents of these compounds and flavonoids were first measured.

At the initial stage, the highest amounts of TPC, 24.5 ± 0.7 mg GAE/g and 24.4 ± 0.4 mg GAE/g, were determined in the BP samples stored at −20 °C and −80 °C, respectively, while the lowest amount, 23.3 ± 0.4 mg GAE/g, was found in the dried BP samples. However, no statistically significant differences were found between these samples (*p* > 0.05).

**Figure 1:**
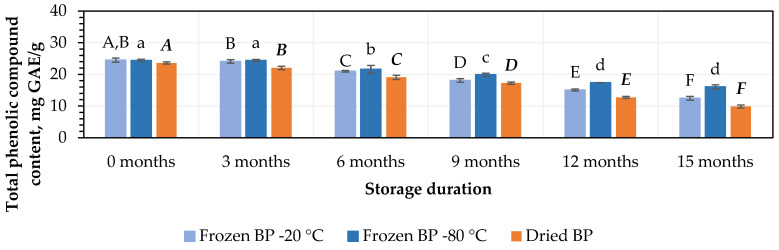
Variation in the amount of total phenolic compounds in bee pollen samples. Different letters indicate statistically significant differences between sample storage durations under the same conditions across different storage periods: −20 °C—uppercase letters; −80 °C—lowercase letters; dried—uppercase letters in bold italics (*p* < 0.05). Assessments were conducted every 3 months over a 15-month period.

The amount of TPC decreased under different storage conditions and over the storage period. During storage (after 3 months), the amount of TPC decreased when the BP samples were stored at temperatures of −20 °C and −80 °C. Meanwhile, a statistically significant (7%, *p* < 0.05) decrease in the TPC was established in the dried BP samples (Figure 1). In the next stage of the storage studies, after 6, 9, 12, and 15 months of storage at −20 °C, the amount of TPC in the BP samples decreased by 14%, 26%, 39%, and 49%, while at −80 °C, the decreases were 12%, 19%, 29%, and 34%, respectively (Figure 1). When analyzing the trends in the changes in TPC in the dried BP samples after 6, 9, 12, and 15 months of storage, the amount of TPC decreased by 10%, 27%, 46%, and 58%, respectively (Figure 1). After the completion of the storage period (after 15 months), the TPC content was 1.2–1.7-fold lower in the dried BP samples compared to that in the frozen BP samples.

The findings of this study revealed that the storage duration had an impact on the content of TPC in both the frozen and dried BP samples. It was found that in the BP samples stored at −20 °C and −80 °C, the content of TPC significantly decreased after 6 months of storage (*p* < 0.05). In the dried BP samples, the TPC content significantly decreased after 3 months of storage (*p* < 0.05) and gradually declined over the entire 15-month period.

**Figure 2:**
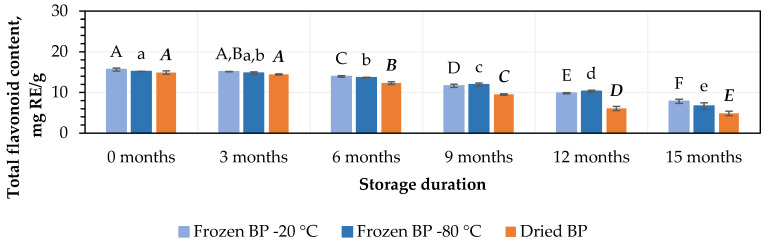
Variations in the total flavonoids in the bee pollen samples. Different letters indicate statistically significant differences between sample storage durations under the same conditions across different storage periods: −20 °C—uppercase letters; −80 °C—lowercase letters; dried—uppercase letters in bold italics (*p* < 0.05). Assessments were conducted every 3 months over a 15-month period.

The total flavonoid content in the BP samples ranged from 14.9 ± 0.5 mg RE/g to 15.7 ± 0.4 mg RE/g (Figure 2). No statistically significant changes were found between the BP samples frozen at different temperatures (−20 °C and −80 °C). The total flavonoid content in the dried BP samples was 4.5% lower than in the frozen (−20 °C) BP samples, but this difference was statistically insignificant.

During this study, the total flavonoid content decreased over the storage period in both the frozen and dried BP samples. After 3, 6, 9, 12, and 15 months of storage at −20 °C, the total flavonoids in the BP samples decreased by 6%, 101%, 26%, 37%, and 50%, while at −80 °C, the decreases were 3%, 10%, 21%, 32%, and 56%, respectively (Figure 2). In the dried BP samples, after 3, 6, 9, 12, and 15 months of storage, the total flavonoids diminished by 3%, 17%, 36%, 59%, and 67%, respectively (Figure 2).

The data from this study revealed that the total flavonoid content remained stable for 3 months in both the dried and fresh frozen BP samples (*p* < 0.05), followed by a steady decline over the subsequent storage period.

### 3.2. Changes in Ascorbic Acid Contents in Bee Pollen Samples

**Figure 3:**
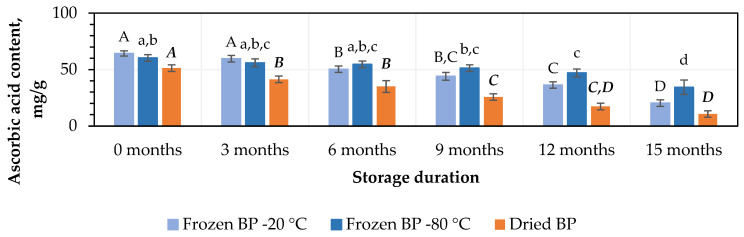
Variations in the amount of ascorbic acid in the bee pollen samples. Different letters indicate statistically significant differences between sample storage durations under the same conditions across different storage periods: −20 °C—uppercase letters; −80 °C—lowercase letters; dried—uppercase letters in bold italics (*p* < 0.05). Assessments were conducted every 3 months over a 15-month period.

At the initial stage, the highest ascorbic acid content, 64.3 ± 2.3 mg/g, was found in the frozen (−20 °C) BP samples, while a lower amount, 51.3 ± 2.9 mg/g, was observed in the dried BP samples (Figure 3). No statistically significant change was found between the BP samples frozen at different temperatures (−20 °C and −80 °C), and the total ascorbic acid contents in the dried BP samples were 15% and 20%, respectively, lower than in the frozen ones (*p* < 0.05).

After 3 months of storage, the level of ascorbic acid decreased by 7% in the frozen BP samples, but no statistically significant difference was determined. In contrast, in dried BP samples, the content of ascorbic acid decreased statistically significantly by 20% (*p* < 0.05) (Figure 3). After 6, 9, 12, and 15 months of storage, the ascorbic acid content decreased most significantly in the dried BP samples, with reductions of 32%, 50%, 66%, and 79%, respectively. In the samples stored at −20 °C, the losses were also considerable—22%, 32%, 44%, and 69%—while storage at −80 °C resulted in comparatively lower decreases of 9%, 15%, 22%, and 43%, respectively (Figure 3). It was found that over the 6–15-month period, the ascorbic acid content in the dried BP samples decreased 1.8–3.4-fold more than in the frozen BP samples stored at −80 °C.

When evaluating different storage durations, the highest amount of ascorbic acid remained when the BP samples were stored at −80 °C. A statistically significant decrease was observed only after 9 months (*p* < 0.05). Meanwhile, in the BP samples stored at −20 °C, a statistically significant decrease was observed after 6 months (*p* < 0.05), whereas in the dried samples, the decline started as early as 3 months (*p* < 0.05) and continued progressively throughout the remaining storage period.

### 3.3. Quantitative and Qualitative Determination of Phenolic Compounds in Bee Pollen Samples

When studying the variation in the qualitative and quantitative composition of phenolic compounds in the BP samples using ultra-efficient liquid chromatography, 25 phenolic compounds were determined (5 phenolic acids and 20 flavonoids).

**Figure 4:**
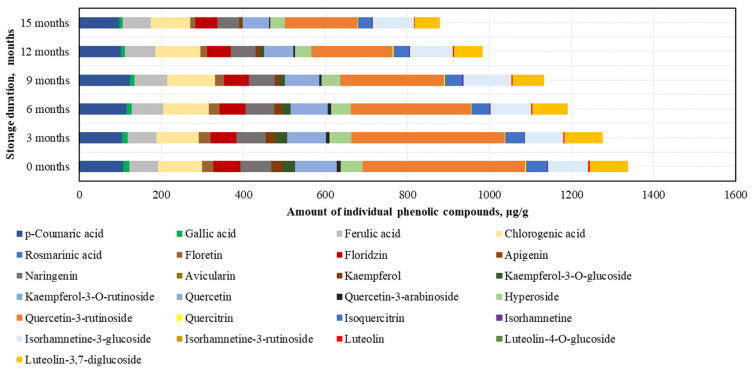
Variations in the contents of the individual phenolic compounds in the frozen (−20 °C) bee pollen samples.

After conducting the analysis of individual phenolic compounds, it was determined that in the BP samples stored at −20 °C, the highest amounts were of rutin (395.4 ± 47.6 μg/g), accounting for 30% of the sum of individual phenolic content (SIPC). High levels of *p*-coumaric acid (106.7 ± 18.3 μg/g), chlorogenic acid (106.4 ± 12.0 μg/g), and quercetin (101.3 ± 5.7 μg/g) were also identified, while the lowest amounts were found for avicularin (0.4 ± 0.04 μg/g), isorhamnetin (2.6 ± 0.1 μg/g), and quercitrin (2.6 ± 0.1 μg/g) (Figure 4).

After 15 months of BP sample storage at −20 °C, changes in phenolic compounds were determined (increasing order): isorhamnetin (+7%) > isorhamnetin-3-glucoside (+3%) > ferulic acid (−3%) > chlorogenic acid (−9%) > *p*-coumaric acid (−9%) > phloridzin (−18%) > naringenin (−28%) > hyperoside (−33%) > luteolin-3,7-diglucoside (−34%) > quercetin (−36%) > isoquercitrin (−37%) > gallic acid (−46%) > quercitrin (−48%) > rutin (−55%) > phloretin (−58%) > luteolin (−64%) > kaempferol (−75%) > quercetin-3-arabinoside (−76%) > kaempferol-3-O-glucoside (−97%) > avicularin (0%) (Figure 4). After 15 months, avicularin was no longer detected. The changes in phenolic compounds occurred gradually over time, with the exception of avicularin, kaempferol, quercetin-3-rutinoside, and kaempferol-3-O-glucoside (which showed a 1.2–2.3-fold decrease over the 6–15 month period), gallic acid, luteolin, and quercetin-3-arabinoside (which showed a 1.2–1.4-fold decrease over the 6–9 month period), and quercitrin (which showed a 1.3-fold decrease over the 9–12 month period).

**Figure 5:**
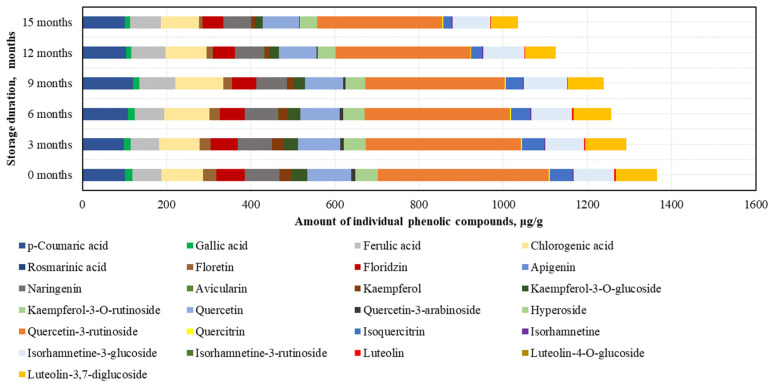
Variation in content of the individual phenolic compounds in frozen (−80 °C) bee pollen samples.

When evaluating the changes in individual phenolic compounds in fresh frozen BP samples stored at −80 °C, it was found that the highest amounts were of rutin (405.5 ± 29.5 μg/g, accounting for 30% of SIPC) and quercetin (104.4 ± 5.3 μg/g), while the lowest amounts were of avicularin (0.4 ± 0.09 μg/g) and quercitrin (2.6 ± 0.2 μg/g) (Figure 5).

After 15 months of fresh frozen BP sample storage at −80 °C, changes in phenolic compounds were observed (increasing order): ferulic acid (+6%) > *p*-coumaric acid (+1%), isorhamnetin-3-glucoside (−8%) > chlorogenic acid (−9%), quercetin (−17%) > naringenin (−20%) > isorhamnetin (−21%) > hyperoside (−22%) > quercitrin (−25%) > rutin (−27%) > phloridzin (−27%) > luteolin-3,7-diglucoside (−33%) > gallic acid (−34%) > kaempferol-3-O-glucoside (−53%) > luteolin (−57%) > isoquercitrin (−63%) > kaempferol (+68%) > phloretin (−72%) > quercetin-3-arabinoside (−85%) > avicularin (−97%) (Figure 5). All identified phenolic compounds were preserved at higher or lower levels in the BP samples stored at −80 °C. After 15 months, the most significant decreases in flavonoid levels in the frozen (−80 °C) BP samples were observed for quercetin-3-arabinoside and avicularin. The changes in phenolic compounds were generally gradual, with the exception of luteolin (a significant 1.3-fold decrease after 3 months), quercetin-3-arabinoside (a significant 5.3-fold decrease during the 6–15 month period), avicularin and phloretin (a significant 1.7–14.8-fold decrease during the 9–15 month period), isoquercitrin (a significant 1.5-fold decrease during the 9–12 month period), and isorhamnetin (a 1.4-fold decrease during the 12–15 month period).

**Figure 6:**
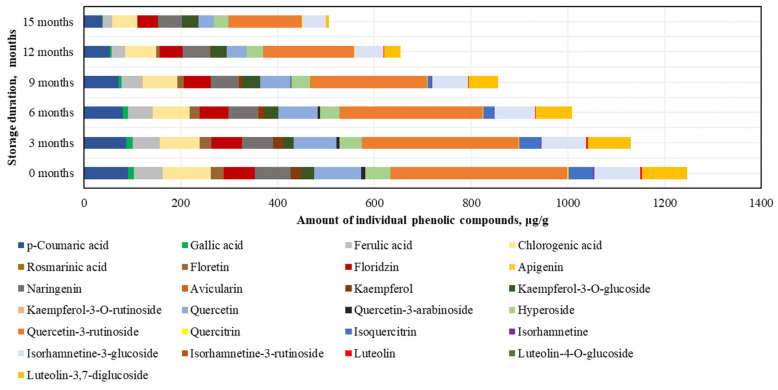
Variations in the contents of the individual phenolic compounds in the dried bee pollen samples.

In the phenolic compound analysis of the dried BP samples, as in the frozen BP samples, the predominant compound was rutin. The highest content of rutin was found in the dried BP samples (366.0 ± 49.8 μg/g, accounting for 29% of SIPC), and the lowest amounts were detected for avicularin (0.3 ± 0.01 μg/g) and isorhamnetin (2.3 ± 0.2 μg/g) (Figure 6).

After 15 months of storage, changes in the phenolic compounds in the dried BP samples were established (increasing order): avicularin (+36%) > kaempferol-3-O-glucoside (+31%) > naringenin (−33%) > phloridzin (−34%) > hyperoside (−41%) > chlorogenic acid (−48%) > isorhamnetin-3-glucoside (−49%) > rutin (−59%) > *p*-coumaric acid (−59%) > quercetin (−67%) > ferulic acid (−68%) > gallic acid (−84%) > quercitrin (−87%) > luteolin-3,7-diglucoside (−94%) > phloretin (−99%) > luteolin (0%) > kaempferol (0%) > isorhamnetin (0%) > isoquercitrin (0%) > quercetin-3-arabinoside (0%) (Figure 6). It is important to note that after 12 months, isorhamnetin, isoquercitrin, and quercetin-3-arabinoside were no longer detected, and after 15 months, luteolin and kaempferol were also absent. Reviewing the results of the dried BP samples, it can be concluded that, compared to the frozen BP samples, the levels of most compounds decreased significantly after 3 months. The greatest quantitative changes were observed in the analysis of kaempferol, quercetin-3-arabinoside, quercitrin, isoquercitrin, isorhamnetin, phloretin, and luteolin.

By evaluating the changes in individual phenolic acids and flavonoids under different storage conditions and durations, the highest total amount of identified phenolic compounds (9105.7 ± 170.8 μg/g) was determined in the frozen BP samples stored at −80 °C (which showed the least variation during storage), while the lowest amount (6961.5 ± 151.7 μg/g) was observed in the dried pollen samples (*p* < 0.05). The fresh frozen (−20 °C) BP samples contained 8509.3 ± 224.2 μg/g. The phenolic compounds identified in the BP samples, in decreasing order, were: rutin > chlorogenic acid > quercetin > *p*-coumaric acid > isorhamnetin-3-glucoside > luteolin-3,7-diglucoside > naringenin > ferulic acid > phloridzin > hyperoside > isorquercitrin > kaempferol-3-O-glucoside > phloretin > kaempferol > gallic acid > quercetin-3-arabinoside > luteolin > isorhamnetin > quercitrin > avicularin. When analyzing the variation in the contents of individual phenolic compounds, the amounts of phloridzin, naringenin, and hyperoside showed the least change during storage, whereas avicularin in the frozen samples decreased most significantly. The results of this study showed that the profiles of BP samples, including complex and individual phenolic compounds, varied significantly under different storage conditions and durations.

### 3.4. Evaluation of the Antiradical and Reducing Activity of Bee Pollen Extracts In Vitro

A review of the literature has shown that both antiradical (ABTS) and reducing (CUPRAC, FRAP) in vitro assays are used for evaluating the antioxidant activity of BP extracts. Different biologically active compounds exhibit distinct antioxidant mechanisms, which may lead to varying results. To ensure accuracy, it is essential to apply multiple methods for evaluation. Considering this possibility and recognizing that using two different methods might result in significant variations and complicate the interpretation of the data, it was decided to employ three different methodologies.

The in vitro antiradical activity values of the BP extracts ranged from 76.5 ± 9.8 μmol TE/g in the frozen (−80 °C) BP extracts to 79.1 ± 0.1 μmol TE/g in the dried BP extracts. No statistically significant differences between the values were found (*p* > 0.05).

**Figure 7:**
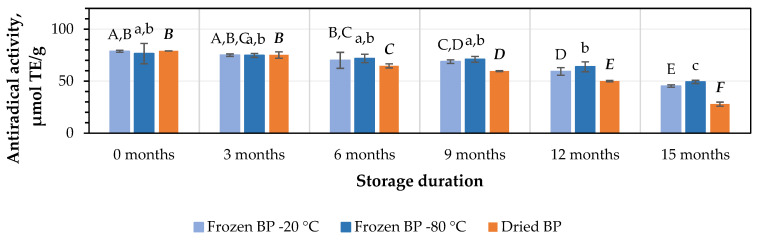
Variations in the antiradical activity in the bee pollen samples determined using the ABTS method in vitro. Different letters indicate statistically significant differences between sample storage durations under the same conditions across different storage periods: −20 °C—uppercase letters; −80 °C—lowercase letters; dried—uppercase letters in bold italics (*p* < 0.05). Assessments were conducted every 3 months over a 15-month period.

After 3 months of storage at −20 °C and −80 °C, the antiradical activity of the BP extracts decreased by 5% and 2%, respectively, while the dried BP extracts showed a slightly higher decrease of 5%. However, these results were statistically insignificant (Figure 7). After 6, 9, 12, and 15 months of storage under different conditions, the antiradical activity of the BP extracts changed as follows: for the frozen BP samples stored at −20 °C, the activity of the BP extracts decreased by 11%, 13%, 25%, and 43%; for the frozen samples stored at −80 °C, the decreases were 6%, 7%, 17%, and 36%; and for the dried samples, the decreases were 18%, 25%, 37%, and 65%, respectively (Figure 7). The research results revealed that the antiradical activity of the frozen (−80 °C) BP extracts decreased slightly less than that of the frozen (−20 °C) and dried BP extracts. Over the 6–15-month period, the antiradical activity of the frozen (−80 °C) BP decreased 1.2–1.8-fold compared to the extracts of the frozen (−20 °C) BP and 1.8–3.4-fold less compared to the dried BP extracts.

A statistically significant decrease in the antiradical activity of the frozen (−80 °C) BP extracts was observed only after 12 months of storage (*p* < 0.05). In the case of the frozen (−20 °C) BP extracts, a statistically significant decrease was observed after 9 months (*p* < 0.05), while in the dried BP extracts, a decrease was already noted after 6 months of storage (*p* < 0.05). Thereafter, the antiradical activity progressively decreased throughout the 15-month storage period.

**Figure 8:**
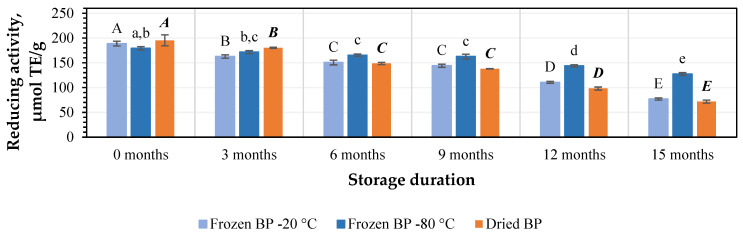
Variations in the reducing activity of the bee pollen samples determined using the CUPRAC method in vitro. Different letters indicate statistically significant differences between sample storage durations under the same conditions across different storage periods: −20 °C—uppercase letters; −80 °C—lowercase letters; dried—uppercase letters in bold italics (*p* < 0.05). Assessments were conducted every 3 months over a 15-month period.

Using the CUPRAC method, the values of the reducing activity capacity in the preliminary stage ranged from 179.2 ± 3.5 μmol TE/g to 195.2 ± 11.0 μmol TE/g. The strongest reducing activity (195.2 ± 11.0 μmol TE/g) was determined in the dried BP extracts, and lower activity (179.2 ± 3.5 μmol TE/g) was found for the frozen (−80 °C) BP extracts (Figure 8). No statistically significant differences between the values in the preliminary stage were found.

Under different storage conditions for 3, 6, 9, 12, and 15 months, the reducing activity of the frozen (−20 °C) BP extracts decreased by 10%, 15%, 20%, 25%, and 36%; the frozen (−80 °C) BP extracts diminished by 7%, 10%, 13%, 17%, and 18%; and the dried BP extracts reduced by 11%, 31%, 32%, 43%, and 56%, respectively (Figure 8). It is noteworthy that after 15 months, the reducing activity of the frozen (−80 °C) BP extracts changed less and remained more stable than the frozen (−20 °C) and dried BP extracts. Over a 6–15-month period, the reducing activity of the frozen (−80 °C) BP extracts decreased 2.6–3.1-fold less compared to the dried BP extracts.

The reducing activity, determined using the CUPRAC method, of the frozen (−80 °C) BP extracts decreased statistically significantly only after 6 months of storage (*p* < 0.05). However, a statistically significant decline in the reducing activity of the frozen (−20 °C) and dried BP extracts was estimated after 3 months (*p* < 0.05). Subsequently, the reducing activity continued to decrease gradually in vitro.

**Figure 9:**
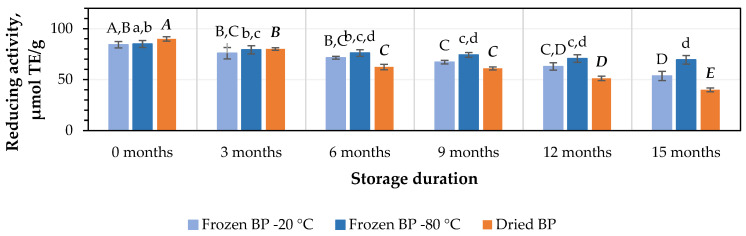
Variations in reducing activity of bee pollen samples determined using the FRAP method in vitro. Different letters indicate statistically significant differences between sample storage durations under the same conditions across different storage periods: −20 °C—uppercase letters; −80 °C—lowercase letters; dried—uppercase letters in bold italics (*p* < 0.05). Assessments were conducted every 3 months over a 15-month period.

The estimation of the reducing potency of the BP extracts in vitro using the FRAP method revealed that this activity ranged from 84.3 ± 3.2 μmol TE/g to 90.0 ± 2.2 μmol TE/g. Similar to the results described earlier, the strongest reducing activity (90.0 ± 2.2 μmol TE/g) was observed in the dried BP extracts, while the lowest activity (84.3 ± 3.2 μmol TE/g) was observed in the frozen (−20 °C) BP extracts (Figure 9). No statistically significant variations between the values in the preliminary stage were found.

The detailed changes in reducing activity during the study, conducted every three months under different storage conditions using the FRAP method, were as follows: for frozen (−20 °C) BP extracts, the reducing activity decreased by 14%, 20%, 24%, 41%, and 59%; for the frozen (−80 °C) BP extracts, it diminished by 4%, 8%, 9%, 20%, and 29%; and for the dried BP extracts, it declined by 8%, 24%, 29%, 50%, and 63% (Figure 9).

When determining the impact of the different storage conditions and durations on the reducing activity of the BP extracts using the FRAP method, it was found that after 3 months of storage, a statistically significant decline in reducing activity was found in the dried BP extracts (*p* < 0.05). In contrast, the reducing activity of the frozen BP extracts, which showed more stable activity, decreased significantly only after 9 months of storage (*p* < 0.05). The reducing activity showed a further gradual decline over 15 months in vitro.

Summarizing the results of the impact of different storage conditions (freezing and drying) and storage duration on the antioxidant capacity of the BP extracts, the strongest antiradical and reducing activity in vitro (over the 6–15-month period) was observed in the frozen (−80 °C) BP extracts compared to the dried BP extracts. During this period, the antiradical and reducing activity in vitro in the frozen BP samples was 1.8–3.4-fold and 2.6–3.1-fold higher than in the dried BP extracts, respectively.

### 3.5. Correlation Analysis

**Figure 10:**
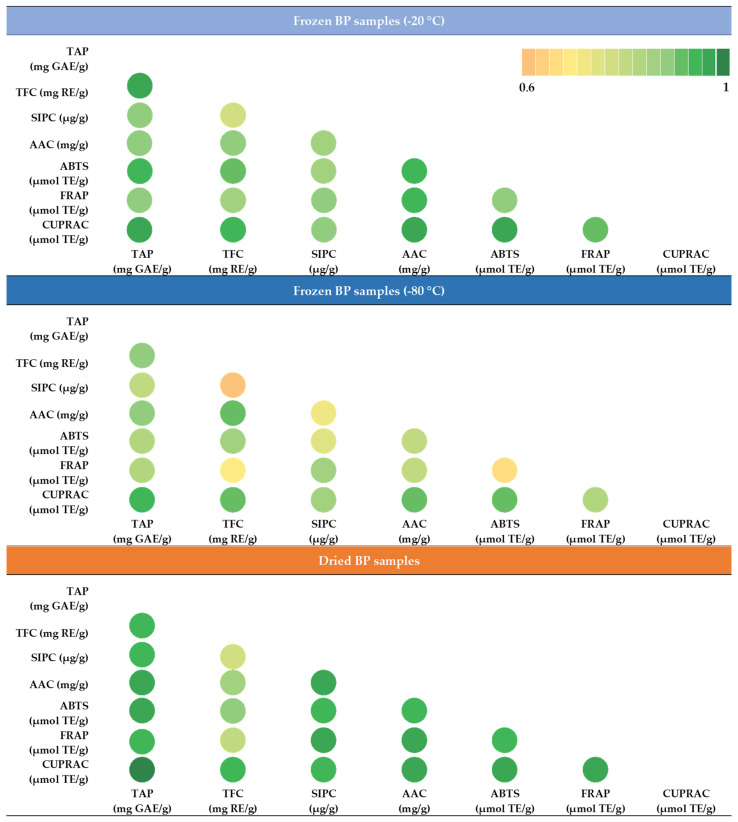
Correlation matrix of the quantitative characteristics of the bee pollen based on Pearson correlation coefficients; different colors indicate the strength of the correlation (*p* < 0.05). Abbreviations: TAP—total amount of phenolic compounds; TFC—total flavonoid content; SIPC—sum of individual phenolic contents; AAC—ascorbic acid content; ABTS—antiradical activity of the BP extracts determined using the ABTS method; FRAP—reducing activity of the BP extracts determined using the FRAP method; CUPRAC—reducing activity of the BP extracts determined using the CUPRAC method.

During this study, the strength of the correlation between the total phenolics content (TAP), sum of individual phenolic compounds (SIPC), total flavonoids (TFC), ascorbic acid (AAC), and the antioxidant activity was determined using the ABTS, FRAP, and CUPRAC methods. Under different conditions (−20 °C, −80 °C, and dried) the stored BP samples were compared and evaluated separately. The matrix created allows for a comprehensive comparison of the correlation relationships between the characteristics selected for evaluation. The intensity of the green color indicates the strength of the correlation (Figure 10).

In the frozen (−20 °C) BP samples, a statistically significant, very strong correlation was observed between the reducing activity measured using the CUPRAC method and TAP (r = 0.969, *p* < 0.05), TFC (r = 0.948, *p* < 0.05), and AAC (r = 0.966, *p* < 0.05). Similarly, a very strong correlation was established between the antiradical activity measured using the ABTS method and TAP (r = 0.940, *p* < 0.05), TFC (r = 0.916, *p* < 0.05), and AAC (r = 0.940, *p* < 0.05). In the frozen (−80 °C) BP samples, only two very strong correlations were found: between the reducing activity determined using the CUPRAC method and TAP (r = 0.944, *p* < 0.05) and ACC (r = 0.911, *p* < 0.05). In the dried BP samples, four statistically significant very strong correlations were found between the reducing activity determined using the CUPRAC method and TAP (r = 0.993, *p* < 0.05), TFC (r = 0.927, *p* < 0.05), SIPC (r = 0.939, *p* < 0.05), and AAC (r = 0.963, *p* < 0.05). Three very strong correlations were also found for the FRAP method and TAP (r = 0.946, *p* < 0.05), SIPC (r = 0.964, *p* < 0.05), and AAC (r = 0.967, *p* < 0.05). Additionally, the ABTS method showed three strong correlations with TAP (r = 0.970, *p* < 0.05), SIPC (r = 0.943, *p* < 0.05), and AAC (r = 0.949, *p* < 0.05).

### 3.6. Botanical Origin of Bee Pollen

The botanical composition of the samples selected for flavonoid analysis is shown in Table 2. Melisopalynological pollen analysis showed that the pollen of *Salix* spp. and *Brassica napus* L. dominated in all the samples, constituting 34.3% and 36.8%, respectively. *Acer platanoides* L., Malus *domestica* Borkh., and *Taraxacum officinale* L. pollens accounted for 12.8%, 9.0%, and 5.9%, respectively, which are also considered as important minor pollens according to the study results.

The coefficient of variance (CV) of the botanical origin of the pollen ranged from 1.37% to 17.84% (Table 2). According to statistics, a CV value is acceptable when it ranges between 10 and 20% [40].

## 4. Discussion

Bee pollen is used as a valuable food source not only for bees but also for humans, as it contains proteins, lipids, amino acids, carbohydrates, vitamins, minerals, and biologically active compounds such as enzymes, phenolic compounds, etc. [20]. The composition of BP varies depending on its botanical and geographical origin, according to the region, and even according to BP collection time [41]. Phenolic compounds, specifically phenolic acids and flavonoids, are important BP compounds known to be potent antioxidants and crucial non-nutrient elements in BP. The method of collection and storage conditions of BP has a significant influence on its phytochemical composition and quality. BP is used by humans not only in its native state but also in the pharmaceutical form (tablets, pastilles etc.), as a food supplement, and in functional food due to biologically active compounds. Consequently, it is particularly essential to choose the right method of BP preparation and its storage conditions. To consumers in Lithuania, dry BP is usually provided, but its quality is highly dependent on the drying temperature. Unfortunately, after drying of pollen, especially at higher temperatures, the amount of biologically active compounds can change dramatically, i.e., decrease, and vitamins are particularly sensitive to high temperatures. Pollen that is freshly collected and frozen immediately after collection is very popular in the foreign market, especially in Romania and other countries, which have long-lasting traditions of usage of BP. Therefore, it is necessary to investigate whether dried or frozen pollen is more valuable, and what optimal storage conditions are necessary, particularly by identifying when the decline in biologically active compounds begins, in order to optimize the amount of biologically active substances in it. Phenolic compounds, particularly phenolic acids and flavonoids, are key bioactive components in BP, recognized for their strong antioxidant properties and essential role in functional foods and pharmaceuticals. While previous studies have highlighted the influence of processing and storage conditions on BP quality, there remains a critical knowledge gap regarding the precise degradation dynamics of polyphenols and vitamin C over time. Our study specifically addresses this gap by systematically investigating the long-term changes in the phytochemical profile of dried and frozen BP under different storage conditions, focusing on when significant reductions in polyphenol and vitamin C content begin. The objective of our study was not to evaluate oxygen-restricted conditions, but rather to examine the stability of bioactive compounds in BP under storage conditions that reflect common consumer and producer practices, namely drying and freezing without specialized packaging. By analyzing these changes at three-month intervals over a 15-month storage period, the first detailed timeline of antioxidant compound degradation in BP was established, offering crucial insights into its optimal preservation. Furthermore, our study provides a comprehensive analysis of the variations in the amount of individual phenolic acids and flavonoids during storage over a 15-month period, offering detailed insights into their stability and degradation dynamics over time. To the best of our knowledge, no such detailed studies have been conducted.

The main findings are as follows: (1) It was determined that during storage (over a 3–15-month period), the total phenolic compound contents began to statistically significantly decrease after 3 months in the dried BP samples, while in the frozen BP samples, this decrease occurred after 6 months (*p* < 0.05). (2) The highest ascorbic acid content remained in the frozen (−80 °C) BP samples, with a statistically significant decrease in the content observed only after 9 months. In the frozen (−20 °C) BP samples, the ascorbic acid content decreased after 6 months (*p* < 0.05), while in the dried BP samples, it decreased after 3 months of storage (*p* < 0.05). (3) The qualitative and quantitative composition of the individual phenolic compounds changed during the 3–15-month period. The content of phloridzin, naringenin, and hyperoside changed the least during storage, while avicularin decreased most significantly in the frozen BP samples (by 100%). (4) The strongest antiradical and reducing activity in vitro (over a 6–15-month period) was observed in the frozen (−80 °C) BP extracts compared to the dried BP extracts and was 1.8–3.4-fold and 2.6–3.1-fold higher, respectively.

Phenolic compounds are among the most widespread secondary metabolites produced in all plant cells [42,43]. In BP, their contents range from 3% to 5% and are highly dependent on the origin of the raw material [44]. The total amount of phenolic compounds in all the tested samples from the BP collected in Lithuania varied from 23.3 ± 0.4 mg GAE/g to 24.5 ± 0.7 mg GAE/g and was lower than that described for Anzer pollens from Turkey (44.1–124.2 mg GAE/g) [45] and pollens from Brazil (41.5–213.2 mg GAE/g) [46]. However, it was similar or higher than that described for Turkish pollen samples (7.9 mg GAE/g and 17.5 mg GAE/g) [47], Italian pollen samples (13.5–24.8 mg GAE/g) [48], Romanian pollen samples (4.4–16.4 mg GAE/g) [49], and Portuguese pollen samples (10.5–16.8 mg GAE/g) [50]. Likewise, the amounts of flavonoids in our BP samples varied from 14.9 ± 0.5 mg RE/g to 15.7 ± 0.4 mg RE/g; however, in other studies, it varied between 4.5 and 7.1 mg CAE/g, 1.8 and 4.4 mg/g QE/g [51], and 1.4 and 9.1 mg QE/g [52]. The total phenolics and total flavonoid contents of twenty-two BP samples from Portugal were determined as 12.9–19.8 mg GAE/g and 4.5–7.1 mg CAE/g, respectively [53].

The most abundant phenolic acids in BP samples are chlorogenic, gallic, ferulic, cinnamic, and caffeic acids, as well as hydroxycinnamic, *o*-coumaric and *p*-coumaric acids [44]. We also confirmed that the major phenolic acids in the BP samples were chlorogenic and *p*-coumaric acids. Flavonoids constituted the most significant group of phenolic compounds present in the BP. The main flavonols in BP are quercetin and kaempherol and their glycosides [44], which was also confirmed in our study. Straumite et al. showed that in BP samples collected in Latvia in 2022 (May and June), the total phenolic contents of polyfloral pollen samples varied from 16.1 ± 0.5 mg GAE/g to 26.1 ± 0.2 mg GAE/g, so the amounts determined were similar to those in our studies [12]. This may have been influenced by the similar geographical location, similar vegetation, climate zone, and time of collection. Meanwhile, research by other scientists (Muliuolytė et al.) shows that in BP samples collected and dried in 2022 in June–August from the apiary located in the south region of Lithuania, the total amount of phenolic compounds varied from 42.1 ± 3.1 mg GAE/g to 93.6 ± 7.3 mg GAE/g, which is higher than in the samples we studied [54]. Such differences could have been affected by differences between seasons, the conditions of extract preparation, different vegetation, weather conditions in that year, and other factors. There have been no studies on how the total amount of phenolic compounds in pollen changes during storage lasting 3 months. In that aspect, our study is new and relevant. However, Stojko et al. investigated three types of BP extracts, namely ethanol extracts, enzymatic hydrolysates from pollen, and ethanol extract of pepsin-digested BP, and showed that 12-month storage of BP extracts decreased phenolic compound concentrations in all three types of extracts, and the changes depended on the storage conditions [44]. The highest decrease in the phenolic compound concentration was in pollen extracts stored at room temperature in light, while storing at 4–8 °C in the dark was the best storage condition for all types of extracts [44]. Anjos et al. investigated the effects of the storage conditions of BP samples from Portugal (frozen at −20 °C or dried at +42 °C) on the quality of BP [27]. They found that even the botanical origin is an important factor in explaining the variation between samples, and the storage method was a highly important factor explaining changes in the amount of total phenolic compounds, total flavonoids, reducing sugars, and lipids [27]. According to Abhay et al., the drying process supports the degradation of phenolic compounds via both enzymatic and non-enzymatic oxidation of phenolic compounds [55]. According to other authors, polyphenol-oxidase enzymes are activated during drying.

Munteanu et al. studied BP samples from nine apiaries in Romania in 2019 [37]. The total amount of flavonoids in frozen BP at the initial stage was determined to be from 2.5 ± 0.3 mg RE/g to 11.8 ± 1.6 mg RE/g, and in dried samples, it was from 2.6 ± 0.04 mg RE/g to 6.9 ± 0.3 mg RE/g. Thus, the total amount of flavonoids was higher in our studied BP samples. The changes after 3 and 6 months in their frozen BP samples were similar to those found in our study, and in dried BP, twice lower amounts of flavonoids were determined, while the total amount of flavonoids in the dried pollen under our chosen conditions after 3 months did not decrease.

By analyzing and comparing the compounds identified during this research in the BP samples, the variations in the phenolic acids profiles were determined. In a study by Turkish scientists [56], caffeic acid and vanillin were detected, and almost twice lower amounts of chlorogenic acid. The abundance of the identified flavonoids in the BP samples was also more diverse. The major compounds were kaempferol, naringenin, and (–)-epicatechin, and the change after 3 months did not differ significantly, as in our studies. The phytochemical composition of BP collected in different regions differs due to different vegetation, different climatic conditions, etc. A study of dried (+30 °C) BP samples performed in Latvia in 2018 showed that the dominant compounds were rutin (428.4 ± 29.1 μg/g), quercetin (127.3 ± 6.2 μg/g), and naringenin (108.9 ± 4.9 μg/g). The results were similar to our research due to similar vegetation in Lithuania, geographical location, and climatic conditions. According to Anjos et al., under favorable storage conditions, higher quantities of biologically active compounds can be extracted from BP samples [27]. This was confirmed by our study results. The levels of *p*-coumaric acid, ferulic acid, chlorogenic acid, isorhamnetin, and isorhamnetin-3-glucoside increased when stored frozen (−20 °C and −80 °C) for 6 and 9 months, while the levels of avicularin and kaempferol-3-O-glucoside increased after 6 and 9 months. After 12 months of storage, the largest growth in the levels of these compounds was detected in dried BP samples, but later, the levels began to decrease. In a study by Anjos et al. of dried BP samples, a significant increase in avicularin (+19%) and naringenin (+11%) was observed after 9 months of storage. Our study results after 9 months showed an increase in avicularin (+26%) and kaempferol-3-O-glucoside (+31%), while naringenin levels decreased during storage (−10%), and after 15 months of storage, it they decreased by almost a third (−33%). Our results are consistent with previous studies. Yousefi et al. reported accelerated degradation of phenolic compounds in freeze-dried berries compared to frozen samples, attributing this to residual enzymatic activity and oxidative processes [57]. Sawicki et al. similarly observed increased flavonoid degradation in freeze-dried apple peels, particularly under elevated storage temperatures [58]. In the context of BP, Komosińska-Vassev et al. and Pascoal et al. demonstrated significant losses of flavonoids during storage at room temperature, which aligned with the trends we observed in our dried samples [6,25]. Furthermore, Almeida-Muradian et al. emphasized that ultra-low temperatures (−80 °C) effectively preserved polyphenolic compounds, supporting our findings regarding the superior stability of frozen BP under such conditions [59].

The amount of ascorbic acid in all the analyzed BP samples ranged from 51.3 ± 2.9 mg/g to 64.3 ± 2.3 mg/g, which is higher than the values reported in other studies, where ascorbic acid content in frozen BP samples collected in Turkey was 42.2 ± 3.4 mg/g, and in dried samples, it was 30.2 ± 2.7 mg/g [5]. Climate zone and vegetation may influence these results. Kolayli et al. investigated the variations in ascorbic acid in frozen (−20 °C) and dried (+30 °C) BP samples, storing the samples under the appropriate conditions for 12 months. After 12 months, in frozen BP samples, ascorbic acid decreased by 27%, and in dried ones, it decreased by 53% [5]. The ascorbic acid content decreased in dried BP samples after 3 months, whereas in fresh-frozen BP samples, no changes were observed after 3 months, and a statistically significant decrease was detected only after 9 months (*p* < 0.05). In fresh-frozen BP samples stored at −20 °C, the ascorbic acid content decreased after 6 months (*p* < 0.05).

We conducted a degradation kinetics analysis using a first-order kinetic model to describe the decrease in total phenolic content, total flavonoid content, and ascorbic acid in bee pollen samples stored under different conditions over 15 months. The kinetic modeling results strongly support the application of a first-order degradation model to describe the storage stability of bioactive compounds in bee pollen. The model provided both mechanistic insights and quantitative parameters: (1) Demonstrate the compound and condition-specific nature of degradation; (2) Confirm that drying accelerates the loss of all tested compounds, especially ascorbic acid and flavonoids; (3) Highlight −80 °C freezing as the most effective strategy for preserving phenolic content, flavonoids, and vitamin C (see Supplementary Material).

The data from our research revealed that the antiradical activity in vitro of the BP extracts varied from 76.5 ± 9.8 μmol TE/g to 79.1 ± 0.1 μmol TE/g. Research has shown that the storage duration can influence the variation in antiradical activity in vitro [60]. The antiradical activity of pollen extracts collected from Brazilian apiaries ranged from 37.6 ± 1.2 μmol TE/g to 70.5 ± 3.3 μmol TE/g, exhibiting weaker antiradical activity compared to the pollen extracts collected under the climatic conditions of Lithuania. After 9 months of storage, the antiradical activity in vitro of our dried BP extracts decreased by 25%, while the Brazilian BP extracts showed a decrease of 17% to 32% [60]. Previous studies have shown that the methanolic extract of BP had a higher ABTS^●+^ radical scavenging capacity (5.37–6.47 TE mg/g RW) compare to bee bread (4.86–5.70 TE mg/g RW) [61]. The results of our investigation demonstrated that the reducing activity in vitro of dried and frozen (−20 °C and −80 °C) BP extracts varied from 179.2 ± 3.5 μmol TE/g to 195.2 ± 11.0 μmol TE/g using the CUPRAC method, and from 84.3 ± 3.2 μmol TE/g to 90.0 ± 2.2 μmol TE/g using the FRAP method. Alimoğlu et al. determined the reducing activity in vitro of frozen (−25 °C) and dried (+30 °C) BP extracts using the CUPRAC method and evaluated the stability of the samples after 12 months [38]. The researchers established that the reducing activity of frozen BP extracts varied from 92.0 ± 8.9 μmol TE/g to 148.6 ± 11.5 μmol TE/g, while for dried BP extracts, it ranged from 89.0 ± 7.6 μmol TE/g to 150.7 ± 9.2 μmol TE/g. According to Turkish scientists, after 12 months of storage, the reducing activity in vitro of frozen BP extracts decreased by an average of 31% (in our study, the decrease was 41%), while in dried extracts, the decrease was 46% on average (in our study, the decrease was 50%). Comparing both studies, it was found that, in the initial stage, the BP extracts examined in this study exhibited stronger reducing activity in vitro; however, compared to the results obtained by Turkish scientists, they were less stable during storage [38]. Croatian scientists carried out research to compare the reducing activity in vitro of BP extracts stored under different conditions (frozen at −20 °C and dried at +30 °C) using the FRAP method [11]. In the initial stage, the reducing activity values of the BP extracts examined in this study were similar to those described by Valentić et al. in their in vitro reducing activity studies (frozen: 105.3 ± 34.1 μmol TE/g; dried: 100.2 ± 7.6 μmol TE/g). According to these researchers, after 9 months, the reducing activity in vitro of frozen BP extracts (−20 °C) was nearly twice as high as that of dried BP extracts (+30 °C) [11]. The results of the Croatian scientist’s study confirm the findings of our research. During our study (over a 6–15-month period), a 2.6–3.1-fold stronger reducing activity in vitro was observed in frozen BP extracts compared to dried extracts.

A statistically significant very strong or strong positive correlation was determined between the content of total phenolic compounds, total flavonoids, the sum of individual phenolic compounds, ascorbic acid, and the in vitro antioxidant activity in all the examined BP samples. The strongest positive correlation was observed between the total phenolic compound content and the reducing activity using the CUPRAC method in dried BP samples (r = 0.993, *p* < 0.05), while in the frozen (−80 °C) BP samples, correlations were observed between the reducing activity using the CUPRAC method and the total phenolic compound content (r = 0.944, *p* < 0.05) and the ascorbic acid content (r = 0.911, *p* < 0.05). Habryka et al. found positive correlations between the content of total phenolic compounds and the total flavonoids (r = 0.9992) and phenolic acids (r = 0.9714) [13]. In many studies, very strong and strong positive correlations were found between the total phenolic compound content of pollen and antioxidant activity (r = 0.930 [62], r = 0.950 [63], r = 0.870 [46]). Therefore, in our study, the relationships described from the literature were observed.

BP is rich in antioxidants, particularly phenolic compounds, the effect of which depends on the presence of double bonds and the positioning of hydroxyl groups on the aromatic ring [64,65]. This ring structure also influences the lipophilicity of polyphenols, particularly flavonoids. Lipophilic antioxidants play a crucial role in protecting lipid membranes [66,67]. Additionally, BP serves as a source of hydrophilic antioxidants, which protect against oxidative damage to cellular structures, including the cytoplasm, organelles, and extracellular fluid. Ascorbic acid and phenolic acids, such as caffeic acid, are examples of hydrophilic antioxidants found in BP. These compounds are effective antioxidants in aqueous phases as well [68]. The antioxidative activity of phenolic acids is influenced by factors such as the number of hydroxyl groups, the arrangement of functional groups, and the steric effects they induce. Monohydroxy derivatives of benzoic acid exhibit the highest antioxidative properties when hydroxylation occurs at the meta position, while dihydroxy derivatives show strong antioxidative activity in both ortho- and meta-hydroxylation. The proximity of the COOH group to ortho-diphenolic functional groups affects the accessibility of hydrogen in the meta position, enhancing antioxidative effectiveness. Gallic acid demonstrates significant antioxidant capacity, equivalent to 3 mM Trolox, due to its three accessible hydroxyl groups. However, the presence of methyl groups at the 3-OH and 5-OH positions reduces its activity compared to trihydroxy derivatives [68]. Monohydroxy derivatives of cinnamic acids are more effective hydrogen donors than those of phenylacetic acid. Furthermore, the introduction of a second hydroxyl group in the ortho position, as seen in caffeic acid, or in the para position, as in protocatechin acid, enhances their antioxidative properties. As a result, diphenols like caffeic, chlorogenic, and protocatechin acids exhibit a stronger ability to scavenge radicals compared to monophenols such as *p*-coumaric acid. Additionally, substituting the 3-OH group with a methoxyl group in caffeic acid, as in ferulic acid, increases antioxidative activity in the lipid phase [69].

Flavonoids’ chemical structure is characterized by a diphenylpropan ring system (C6-C3-C6) with a benzo-γ-pyrone skeleton. The antioxidative properties of flavonoids are influenced by the presence of a double bond between C2 and C3 in the C ring. Additionally, a carbonyl group at the C4 position enables flavonoids to scavenge hydroxyl radicals. The presence of a hydroxyl group at the C3 position in the C ring allows for the inhibition of lipid peroxidation. The ability to scavenge hydroxyl radicals increases with the number of hydroxyl groups present in the B ring, particularly at positions 3′ and 4′. Furthermore, hydroxyl groups at the C5 and C7 positions in the A ring, C3′ and C4′ in the B ring, and C3 in the C ring enhance the inhibition of lipid peroxidation [68,70,71,72,73]. Various flavonoid derivatives have different additions to antioxidant activity [74]. Among quercetin glycosides quercetin 3-O rutoside (rutin) is a common form [75,76]. Its sugar component consists of a glucose and rhamnose disaccharide. Some researchers suggest that the optimal level of rutin in pollen determines its biological and nutritional quality [75]. Quercetin derivates such as C(3)-OH and C(4′)-OH glycoside showed decreased antioxidant activity compared to its free form, quercetin aglycone (without linked sugars) [77]. However, it is claimed that supplements containing quercetin in the aglycone form are less bio-absorbed by the body than quercetin glycoside, which is often found in food [78,79]. Liangqin Xie et al. determined that the antioxidant activity of flavonoid C-glycosides is equal to or greater than that of O-glycosides. Glycosides maintain relatively stable antioxidant activity compared to their aglycones. The structural stability differences between flavonoid O-glycosides, C-glycosides, and their aglycones contribute to changes in their content during digestion, resulting in differences in their antioxidant activity. The antioxidant activity of flavonoid O-glycosides in plasma was higher than that of C-glycosides, while in urine, C-glycosides exhibited higher activity than O-glycosides, reflecting their distinct absorption and metabolism pathways [80].

*Apis mellifera* honeybees can detect floral scents to locate and distinguish different flowers while searching for food. Studies show that both odor intensity and quality affect bees’ ability to distinguish floral scents [81]. Rapeseed is very attractive to insect pollinators due to the rich yellow color of its flowers, its special aromas, and the sugar content in its nectar [82,83]. Semi-natural habitats in the landscape have a positive effect on the diversity of pollen collected by bees [84]. Pollen from flowers of different plants has a specific size, shape, and exine surface ornamentation. The investigation of honey plant pollen is of great importance for the identification of floral resources of nectar-producing plants [28]. Monofloral and polyfloral honey is collected in Lithuania, as well as monofloral or polyfloral pollen and bee bread [85,86,87]. Data show that in Lithuania, beekeeping products are mainly collected from rapeseed, but monofloral honey is also collected from apple trees (*Malus domestica*, Borkh.), linden (*Tilia cordata* Mill.), caraway (*Carum carvi* L.), white clover (*Trifolium repens* L.), and willow (*Salix* spp.) [88].

Our data show that in the investigated samples, Salix spp. and *Brassica napus* L. were the dominant pollen types, comprising 34.3% and 36.8% of the total, respectively. Additionally, *Acer platanoides* L., *Malus domestica* Borkh., and *Taraxacum officinale* L. were identified as significant minor pollen contributors, accounting for 12.8%, 9.0%, and 5.9% of the composition, respectively. Baltrušaitytė, V. et al. studied the botanical composition and antioxidant activity of 35 Lithuanian honey samples and 9 samples of bee bread. The strongest (DPPH^•^) radical scavenging, which was greater than 80%, was found for 4 samples out of 35, including monofloral spring rape, willow, and polyfloral spring honey [89].

The biologically active compounds present in BP, such as flavonoids and phenolic acids, exhibit a broad range of biological activities, with their effects potentially varying depending on the stability of these compounds during storage. The degradation of ascorbic acid and phenolic compounds in BP during storage can be influenced by several factors, including oxygen, light, and temperature. Enzymatic activity, particularly that of polyphenol oxidase, may contribute to the degradation of phenolic compounds if enzyme inactivation is incomplete, especially in dried pollen [25]. A key pathway involves enzymatic oxidation, in which polyphenol oxidases catalyze the conversion of ortho-diphenols to highly reactive quinones. These quinones can subsequently participate in non-enzymatic polymerization reactions, forming brown pigments such as melanins. This process not only alters the visual appearance of the product but may also lead to a reduction in antioxidant activity, thereby affecting its functional quality [90]. It can also affect another mechanism, hydrolysis of flavonoid glycosides, which can occur in the presence of β-glucosidases, resulting in the release of aglycones. Flavonoid glycosides are subject to enzymatic hydrolysis, and their stability depends on both the sugar moiety and the position of glycosylation. In a study by Zhang et al., quercetin 3-O-glucoside was hydrolyzed most efficiently by β-glycosidase from propolis, while glycosides containing disaccharides such as rutin and naringin remained stable and resistant to enzymatic cleavage. These findings highlight that mono-glucosides are more susceptible to degradation, which may influence the overall bioavailability and antioxidant activity of flavonoid-rich products during processing or storage [91]. Temperature is another factor that can accelerate degradation. Heat during drying may cause thermal decomposition of flavonoids and vitamin C, while even frozen samples are susceptible to slow oxidative processes over extended periods. To maximize the retention of vitamin C, polyphenols, and antioxidant properties in BP, freezing (−20 °C) is the most effective method, while drying at 40 °C results in significant degradation of these bioactive compounds. High-pressure processing emerges as a promising alternative to preserve bioactive compounds while ensuring microbiological safety [92,93]. Freezing (−20 °C) is the most effective method to preserve vitamin C in BP, as drying at 40 °C causes significant losses, with up to 90% degradation after 12 months of storage [94]. The antioxidant potential of BP decreases over time, particularly at room temperature, with freeze-drying and high-pressure processing preserving flavonoids and polyphenols more effectively than hot air drying [92]. Fresh pollen contains higher levels of polyphenols, flavonoids, and amino acids than dried pollen, with drying at 40 °C reducing these bioactive compounds, while freeze-drying and microwave-assisted drying better preserve them [95]. Storage conditions greatly influence the stability of bioactive compounds, with freezing (−20 °C) significantly slowing polyphenol degradation, whereas room temperature storage and light exposure accelerate their loss [93,94]. Rutin and other flavonoids degrade more rapidly in hot air-dried pollen compared to freeze-dried or microwave-dried pollen, with high-pressure processing (HPP) emerging as a promising alternative for preserving these compounds [92,95]. Although our study did not investigate these mechanisms in detail, the observed trends are consistent with known degradation pathways reported in the literature. Future research should explore the stability of individual compounds and the specific mechanisms through which environmental factors influence their degradation. To maintain the stability of phenolic compounds and preserve the biological activity of BP during storage, it is essential to consider the influence of environmental factors [14,27,96]. It is recommended that BP be stored at low temperatures, such as in a refrigerator (approximately 4 °C) or in a freezer (−20 °C), in order to minimize the risk of oxidation of phenolic compounds. Exposure to higher temperatures is known to accelerate the degradation of these active components. The stability of flavonoids during storage can be influenced by both light and oxygen, with these factors potentially having a synergistic effect [97]. Scientific studies have shown that short-term exposure of apples to UV radiation stimulates the synthesis of phenolic compounds; however, prolonged exposure leads to their degradation and a decrease in their content. It has also been found that low doses of gamma ionizing radiation break glycosidic bonds of tannins and contribute to an increase in proanthocyanidin and flavan-3-ol levels. The enhanced synthesis of phenolic compounds is a response to photobiological stress induced by light radiation and environmental temperature fluctuations, during which enzymes such as superoxide dismutase, catalase, phenol peroxidase, and others are activated [98]. Flavonoid degradation can occur immediately or be delayed for varying periods depending on the molecular structure and environmental conditions. The photostability of various flavonoids depends on a 3-OH group and is a key factor in flavonoid photoreactivity. Tournaire et al. [99] highlighted that flavonoid reactivity to oxygen depends on the C-ring structure. They found that flavanones and flavanols are chemically stable in the presence of oxygen, while flavones and flavonols exhibit higher sensitivity. Furthermore, the rate of oxygen-induced degradation significantly decreases when the hydroxyl group at position 3 is absent. This can be explained by the fact that the 3-OH group activates the C2–C3 double bond in the presence of oxygen, leading to the opening of the C-ring in the flavonoid structure [100]. Additionally, pollen should be stored in dark containers or sealed bags to protect it from direct UV exposure. Moisture, which promotes enzyme activity and oxidative processes, should be avoided; therefore, BP should be kept in a dry environment to prevent biochemical reactions that may reduce its effectiveness.

In summary, new, important, and significant results were obtained, showing the value and superiority of fresh frozen pollen compared to dried pollen, since the preparation method of dried pollen dominates in Lithuania. Moreover, this study provides novel insights into the dynamic changes in individual compounds, specifically phenolic acids and flavonoids, during storage over a 15-month period with assessments conducted every three months—an aspect that, to the best of our knowledge, has not been previously detailed. The obtained results are important for both beekeepers and consumers in order to know when the amounts of antioxidants, including phenolic compounds and vitamins, start to decrease during storage. This is also very important in the pharmaceutical industry in order to use the highest quality of BP samples for the production of food supplements and pharmaceutical preparations with BP.

## 5. Conclusions

It was determined that during storage (over a 3–15-month period), the total amount of phenolic compounds in dried and frozen (at −20 °C and −80 °C) bee pollen gradually decreased by 7% to 58%, while the total amount of flavonoids decreased by 10% to 67% (*p* < 0.05). The highest total amounts of phenolic compounds (24.4 ± 0.4 mg GAE/g, *p* < 0.05) and flavonoids (15.2 ± 0.07 mg GAE/g, *p* < 0.05) were maintained in the fresh frozen BP samples stored at −80 °C. In the dried BP samples, the total phenolic content started to decrease after 3 months, while in the fresh frozen BP samples, it decreased after 6 months (*p* < 0.05). After UESC-MS analysis, 20 flavonoids (mainly rutin, quercetin, isorhamnetin-3-glucoside, luteolin-3,7-diglucoside, naringenin, and phloridzin) and 5 phenolic acids (mainly *p*-coumaric and chlorogenic acid) were estimated in the BP samples. It was determined that after the 3–15 month-storage period, the highest total amount of identified phenolic compounds (9105.7 ± 170.8 μg/g) was found in fresh frozen BP samples stored at −80 °C (which showed the least variation during storage), while the lowest amount (6961.5 ± 151.8 μg/g) was observed in the dried pollen samples (*p* < 0.05). The qualitative and quantitative composition of individual phenolic compounds changed during the 3–15-month period. The levels of phloridzin, naringenin, and hyperoside changed the least during storage, while avicularin showed the most significant decrease in the frozen BP samples (decreased by 100%). When evaluating different storage conditions and durations, the highest amount of ascorbic acid remained in the frozen BP samples stored at −80 °C (a statistically significant decrease in content was observed only after 9 months, *p* < 0.05). In the frozen BP samples stored at −20 °C, the ascorbic acid content decreased after 6 months (*p* < 0.05), while in the dried BP samples, a decrease was observed after 3 months of storage (*p* < 0.05). The strongest antiradical and reducing activity in vitro (over a 6–15-month period) were established in the frozen (−80 °C) BP extracts compared to the dried pollen extracts. During storage, the antiradical and reducing activities in the frozen (−80 °C) BP pollen extracts were 1.8–3.4-fold and 2.6–3.1-fold higher compared to the dried BP pollen extracts, respectively. Melisopalynological analysis revealed a polyfloral pollen mixture, with *Salix* spp. and *Brassica napus* L. predominating in all the samples, comprising 34.3% and 36.8%, respectively. Additionally, the pollens of *Acer platanoides* L., *Malus domestica* Borkh., and *Taraxacum officinale* L. contributed significantly to the composition.

## Figures and Tables

**Table 1:** Collision energy and cone voltage of phenolic compounds.

Compound	Parent Ion (*m*/*z*)	Daughter Ion (*m*/*z*)	Cone Voltage, V	Collision Energy, eV
*p*-Coumaric acid	163	93	28	22
Gallic acid	169	51	36	30
Ferulic acid	193	134	32	18
Chlorogenic acid	353	191	32	14
Rosmarinic acid	359	161	36	16
Phloretin	273	167	42	16
Phloridzin	435	273	42	14
Apigenin	269	117	54	36
Naringenin	271	151	46	18
Avicularin	433	301	50	20
Kaempferol	285	185	50	25
Kaempferol-3-O-glucoside	447	284	54	28
Kaempferol-3-O-rutinoside	593	285	36	20
Quercetin	301	151	48	20
Quercetin-3-arabinopiranoside	433	300	56	26
Hyperoside	463	300	50	26
Rutin	609	300	70	38
Quercitrin	447	300	50	26
Isoquercitrin	463	301	52	28
Isorhamnetin	315	300	44	22
Isorhamnetin-3-glucoside	477	314	60	28
Isorhamnetin-3-O-rutinoside	623	315	70	32
Luteolin	285	133	58	36
Luteolin-4-O-glucoside	447	285	36	16
Luteolino-3.7-diglucoside	609	447	30	20

**Table 2:** The botanical composition of the samples selected for flavonoid analysis.

Plant Pollen	Average, %	Variation Limits		SD	CV, %
		Min.	Max.		
*Brassica napus* L.	35.37	34.30	36.40	1.05	2.97
*Salix* spp.	36.87	36.4	37.40	0.50	1.37
*Acer platanoides* L.	12.83	11.0	15.40	2.29	17.84
*Malus domestica* Borkh.	9.03	8.10	10.40	1.21	13.39
*Taraxacum officinale* L.	5.93	5.40	6.70	0.68	11.47

Note: standard deviation of the mean (SD); coefficient of variance (CV).

## Data Availability

The data are contained within the article.

## Supplementary Materials

The following supporting information can be downloaded at: https://www.mdpi.com/article/10.3390/antiox14040462/s1, Table S1. Parameters of the first-order degradation model for phenolic compounds in bee pollen samples. Figure S1. First-order degradation model of phenolic compounds in bee pollen samples. Table S2. Parameters of the first-order degradation model for flavonoids in bee pollen samples. Figure S2. First-order degradation model of flavonoids in bee pollen samples. Table S3. Parameters of the first-order degradation model for ascorbic acid in bee pollen samples. Figure S3. First-order degradation model of ascorbic acid in bee pollen samples.
