# Supplementary material for: Changes in Ascorbic Acid, Phenolic Compound Content, and Antioxidant Activity In Vitro in Bee Pollen Depending on Storage Conditions: Impact of Drying and Freezing
Source: Antioxidants (Basel). 2025 Apr 12;14(4):462. doi: 10.3390/antiox14040462 (PMC12024237; doi:10.3390/antiox14040462)
Supplement: Supplementary file 1 [file antioxidants-14-00462-s001.zip › antioxidants-3538081-supplementary.pdf]

The kinetic modeling results strongly support the application of a first-order degradation model to describe the storage stability of bioactive compounds in bee pollen. The model provided both mechanistic insights and quantitative parameters that: 1) Demonstrate the compound and condition-specific nature of degradation; 2) confirm that drying accelerates the loss of all tested compounds, especially ascorbic acid and flavonoids. 3) Highlight -80 °C freezing as the most effective strategy for preserving phenolic content, flavonoids and vitamin C

The degradation of phenolic compounds was modeled using the first-order kinetic equation: $C_t = C_0 \times e^{-kt}$, where:

- C_t is the amount of total phenolic compounds at time t,
- C_0 is the initial content,
- k is the degradation rate constant (month^{-1}),
- t is storage time in months.

Table S1. Parameters of the first-order degradation model for phenolic compounds in bee pollen samples.

**Sample**	**C_0 (mg/g)**	**k (month^{-1})**	**R^2**
Frozen BP –20 °C	24.71	0.0456	0.995
Frozen BP –80 °C	24.53	0.0295	0.992
Dried BP	23.89	0.0571	0.997

As shown in the Table 1 and the Figure 1, the first-order model accurately describes the degradation kinetics of phenolic compounds in all tested conditions ($R^2 > 0.99$). Notably, dried samples exhibited the fastest degradation, whereas samples stored at -80 °C demonstrated the slowest rate, indicating superior preservation of phenolic compounds under deep-freezing conditions. This modeling provides a predictive and mechanistic understanding of compound stability and supports more precise interpretation of storage-related quality loss.

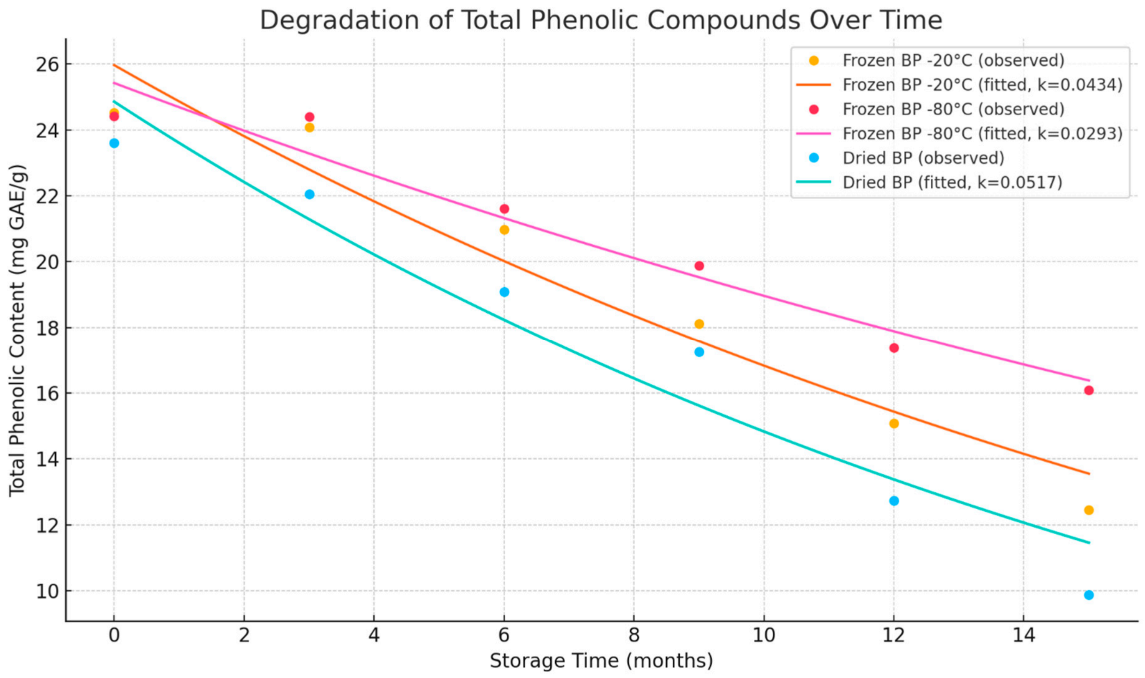

Figure S1. First-order degradation model of phenolic compounds in bee pollen samples.

Regarding flavonoids: we performed a kinetic analysis of the total flavonoid content in dried and frozen bee pollen samples using a first-order degradation model. The degradation kinetics were modeled with the equation: $C_t = C_0 \times e^{-kt}$, where:

- C_t is the amount of flavonoid content (mg RE/g) at time t,
- C_0 is the initial content,
- k is the degradation rate constant ($month^{-1}$),
- t is storage time in months.

Table S2. Parameters of the first-order degradation model for flavonoids in bee pollen samples.

Sample	C_0 (mg/g)	k ($month^{-1}$)	R^2
Frozen BP -20 °C	17.13	0.0455	0.942
Frozen BP -80 °C	17.40	0.0581	0.848
Dried BP	17.83	0.0863	0.934

The model fit was good in all cases, with R^2 values indicating a consistent first-order degradation profile (Table 2, Figure 2). The fastest degradation was observed in dried pollen, while freezing at –20 °C resulted in the slowest flavonoid loss, suggesting this condition provides better preservation than –80 °C, possibly due to matrix-specific freezing behavior and moisture retention. These findings enhance the mechanistic understanding of compound stability and support the predictive value of the study.

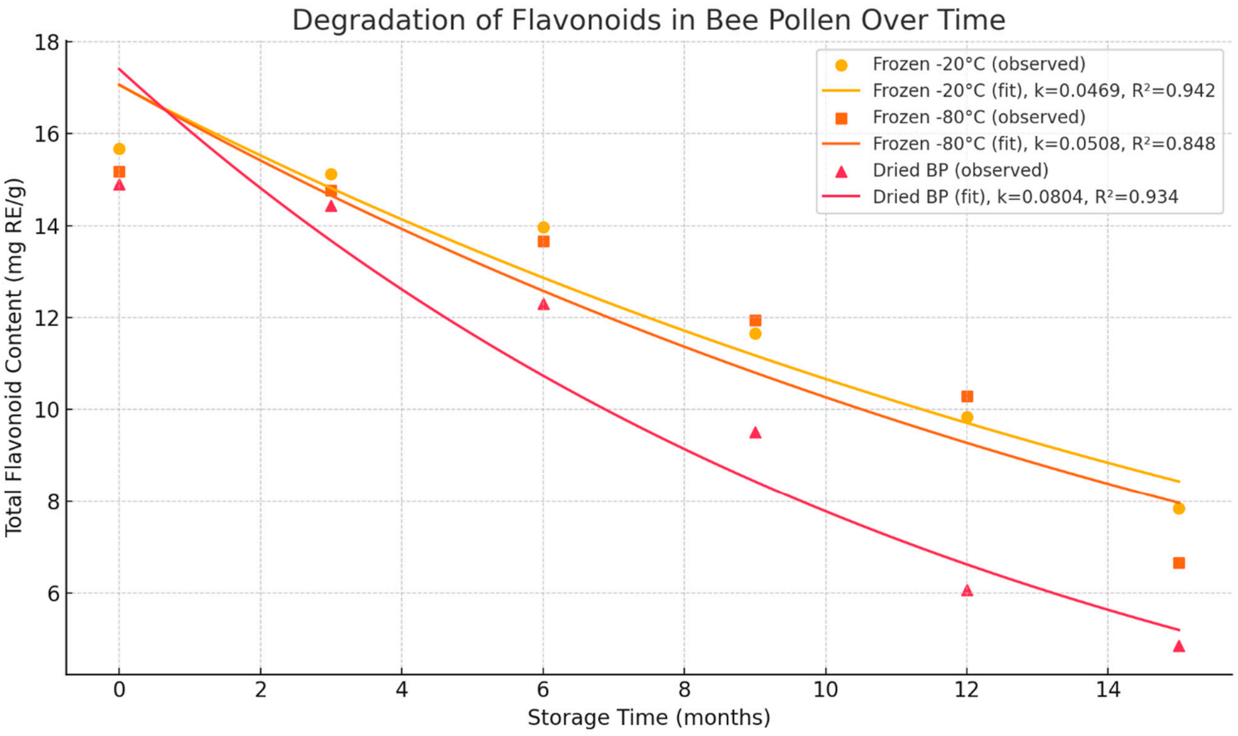

Figure S2. First-order degradation model of flavonoids in bee pollen samples.

Regarding ascorbic acid: In response, we performed a kinetic degradation analysis of ascorbic acid in bee pollen stored under three different conditions over a 15-month period. A **first-order kinetic model** was applied to evaluate the degradation profile, which is commonly used for thermolabile compounds such as ascorbic acid.

The degradation process was modeled using the following equation: $C_t = C_0 \times e^{-kt}$, where:

- C_t is the ascorbic acid content at time t,
- C_0 is the initial content,
- k is the degradation rate constant (month^{-1}),
- t is time in months.
- Natural logarithms of the concentrations were linearly regressed against time to determine the degradation rate constants and assess model fit using the determination coefficient R^2.

Table S3. Parameters of the first-order degradation model for ascorbic acid in bee pollen samples.

**Sample**	**C_0 (mg/ g)**	***k* (month^{-1})**	***R^2***
Frozen BP –20 °C	72.78	0.0703	0.880
Frozen BP –80 °C	63.55	0.0325	0.822
Dried BP	57.37	0.1026	0.963

The degradation of ascorbic acid follows first-order kinetics with good model fit (especially for dried samples) (Table3, Figure 3). The fastest degradation was observed in dried bee pollen, while freezing at –80 °C showed the best preservation of ascorbic acid. This kinetic modeling adds mechanistic insight and predictive value to the interpretation of our data and supports our storage condition comparisons.

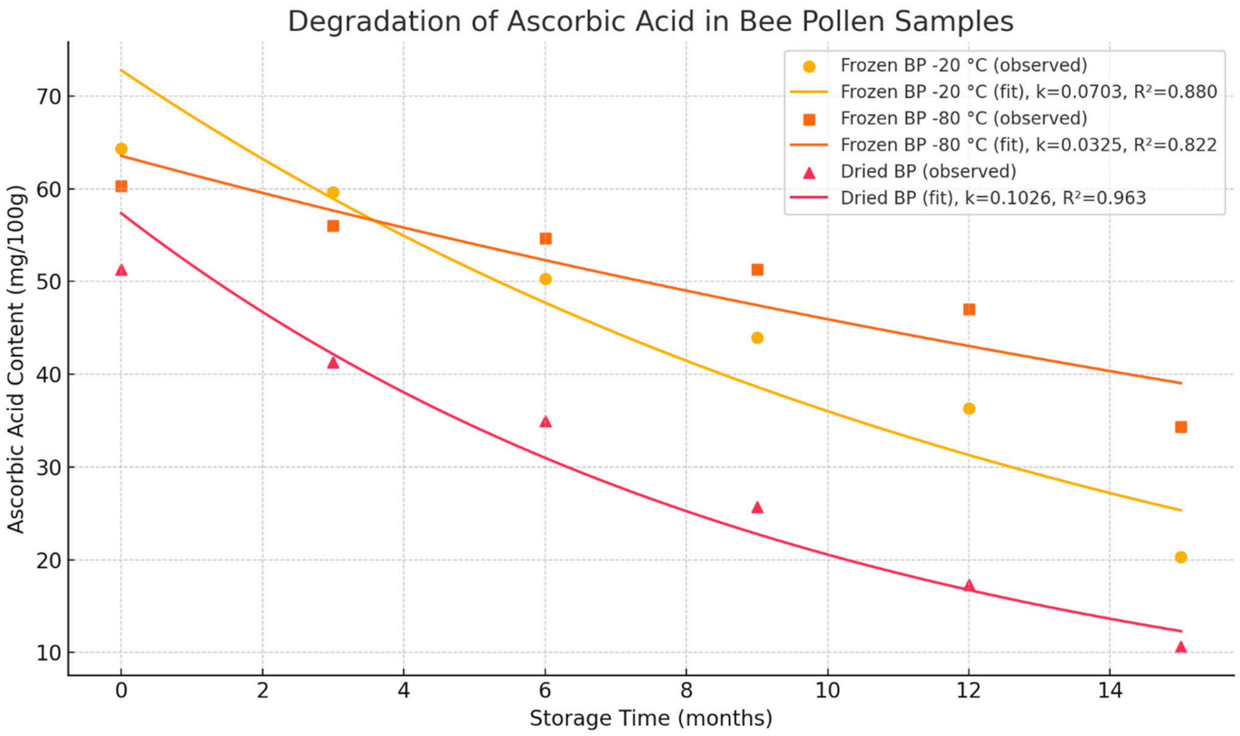

Figure S3. First-order degradation model of ascorbic acid in bee pollen samples
